# A Retrospective Examination of Sleep Disturbance across the Course of Bipolar Disorder

**DOI:** 10.4172/2167-0277.1000193

**Published:** 2015-03-30

**Authors:** Jennifer C Kanady, Adriane M Soehnera, Allison G Harvey

**Affiliations:** 1 Department of Psychology, University of California, Berkeley, Berkeley, CA, USA; 2 Department of Psychiatry, University of Pittsburgh, School of Medicine, Pittsburgh, PA, USA

**Keywords:** Bipolar disorder, Sleep disturbance, Prevalence, Coexistence, Persistence

## Abstract

**Background:**

Sleep disturbance is a prevalent and clinically significant feature of bipolar disorder. However, there are aspects of sleep and bipolar disorder that have been minimally characterized. This study aims to fill several gaps in the literature by examining the prevalence, coexistence, and persistence of sleep disturbance retrospectively across a five-year period in bipolar disorder.

**Methods:**

Fifty-one people with bipolar disorder I and comorbid insomnia who were currently inter-episode completed the NIMH Retrospective Life-Charting Methodology (the life chart). The life chart was used to document the prevalence, coexistence, and persistence of insomnia, hypersomnia, delayed sleep phase, reduced sleep need, and irregular sleep patterns across the course of five years.

**Results:**

Across the five year period, manic months were primarily characterized by reduced sleep need (62.8%) and insomnia (38.1%), depressive months by hypersomnia (56.0%) and insomnia (51.9%), mixed months by all five types of sleep disturbance, and inter-episode months by insomnia (67.4%). There was coexistence in the types of sleep disturbance experienced. Further, each type of sleep disturbance demonstrated persistence across the five years, with persistence rates being the highest for insomnia (49.0–58.8%).

**Conclusions:**

Sleep disturbance is a prevalent and complex feature across mood episodes and inter-episode periods of bipolar disorder. Further, there is variation in the types of sleep disturbance experienced.

## Introduction

Bipolar disorder is a severe disorder characterized by mood episodes; namely, periods of elevated or irritable mood, referred to as mania, periods of depression, and mixed manic and depressed states [1]. Bipolar disorder is further characterized by periods identified as neither depressive nor manic, a phase referred to as the inter-episode period [2–4]. Although there have been important advances in psychological and pharmacological treatments for bipolar disorder, it remains a severe and chronic psychiatric illness with a lifetime prevalence of approximately 1.0% [5] and is one of the 10 most disabling conditions worldwide [6]. Further, bipolar disorder is associated with significant impairment across numerous functional and health domains and this impairment often persists during the inter-episode period [2,3,7–9]. Sleep is one important domain that is often impaired in bipolar disorder and is the focus of the current study.

Sleep disturbance is a common feature of bipolar disorder. Within the Diagnostic and Statistical Manual of Mental Disorders (DSM-5), sleep disturbance is listed as a diagnostic criterion of mania, depression, and mixed episodes [1]. Moreover, sleep disturbance persists during the inter-episode period and as many as 70% of people with bipolar disorder report clinically significant sleep disturbance during inter-episode states [10]. Different types of sleep disturbance characterize bipolar disorder. Insomnia, for example, is highly prevalent [11] with as many as 100% of people with bipolar disorder report experiencing insomnia while depressed [12] and 55% reporting insomnia during the inter-episode period [10]. Episodes of depression are also characterized by hypersomnia [13] and 25% of people endorse symptoms of hypersomnia during the inter-episode period [14]. A reduced need for sleep is often exhibited during mania [15] and mixed episodes are characterized by decreases in total sleep time [16]. Furthermore, people with bipolar disorder are more likely to report circadian dysfunction, such as a delayed sleep phase preference, when compared to healthy individuals, [17,18] and irregular sleep patterns are commonly reported [19–21].

Notably, these different types of sleep disturbance can coexist and sleep disturbance is often persistent. For example, two studies have examined the coexistence of sleep disturbance in the context of depression and found that 10% of children with Major Depressive Disorder [22,23] and 8–11% of older adults in a depressive episode [23] experience insomnia and hypersomnia simultaneously. Moreover, in general population studies, 6% of adults [24] and 8% of young adults [25] also reported comorbid insomnia and hypersomnia. The coexistence of other types of sleep disturbance has yet to be examined. Sleep disturbance is also persistent. Longitudinal studies in healthy, aging, and depressed samples have shown insomnia persistence rates ranging from 40–74% [25–29]. Insomnia relapse rates are high and 25% of individuals who remit report at least one relapse over a 3-year period [30]. Furthermore, individuals with symptoms of insomnia, who do not meet diagnostic criteria for insomnia, are more likely to relapse and report worsening of insomnia over time [30]. Few studies have examined the persistence of other types of sleep disturbance. However, one study found that the lifetime prevalence of hypersomnia alone is 8.2% and the lifetime prevalence of hypersomnia in conjunction with insomnia is 8% [25].

Taken together, it is clear that sleep disturbance is a prevalent and variable feature of bipolar disorder and sleep disturbance may coexist and be persistent. However, a number of unanswered questions remain. For instance, no studies have examined the course or variability of sleep disturbance across several mood episodes and inter-episode periods of bipolar disorder. Previous studies examining sleep disturbance in bipolar disorder have typically focused on a single mood episode or a single period of time. Moreover, previous research has typically focused on just one type of disturbance and therefore, the potential coexistence of different types of sleep disturbance during a single period of time has yet to be determined. A second gap is that the persistence of sleep disturbance across the course of bipolar disorder has yet to be established. Sleep disturbance is a common feature of both mood episodes and the inter-episode period. However, it is unclear if, and how, sleep disturbance endures or changes from year to year.

The goal of the present study was to contribute to the process of closing these important gaps in the literature by examining the course of sleep disturbance across bipolar disorder. The first aim was to retrospectively examine the prevalence and coexistence of insomnia, hypersomnia, delayed sleep phase, reduced sleep need, and irregular sleep patterns across mood episodes and inter-episode periods over the last five years. Based on previous research, we hypothesized that sleep disturbance would be a prevalent feature across this five-year period and that the different types of sleep disturbance would coexist. The second aim was to retrospectively examine the persistence of each type of sleep disturbance across the same five-year period. Based on previous research, we hypothesized that sleep disturbance would be a persistent feature across this same five-year period. Information about the course of the different types of sleep disturbance in bipolar disorder is important for a number of reasons. First, it will pave the way to improving our understanding of this prominent but understudied feature of mood episodes and inter-episode periods. Second, sleep disturbance is a prominent correlate of mood disruption [31–37] and better understanding the course of sleep disturbance across bipolar disorder may aid in the prevention of relapse. Finally, understanding the course of sleep disturbance will help guide the development of more focused interventions.

## Methods

### Participants

The aims of the present study were addressed in the context of an NIMH-funded trial examining the effectiveness of treating insomnia during inter-episode bipolar disorder with cognitive behavior therapy for insomnia [CBT-I; e.g., 38]. Hence, all participants met criteria for bipolar I disorder and current insomnia and were inter-episode at the time of assessment.

Fifty-one people who met DSM-IV-TR criteria [15] for bipolar I disorder were included in the study. Bipolar diagnoses were assessed using the Structured Clinical Interview for Axis I Disorders [SCID; 39]. Ethnicity and race of the participants reflected the diversity of Alameda County (63% Caucasian, 11.1% African American, 9.3% Asian, 9.3% more than one ethnicity, 3.7% declined to answer, 1.9% American Indian/Alaska Native, 1.9% other). Participants were inter-episode (i.e., not currently manic or depressed) at the time of the assessment. An inter-episode period was defined by a score of 24 or less on the Inventory of Depressive Symptomatology, Clinician Rating [IDS-C; 40] and a score of 12 or less on the Young Mania Rating Scale, [YMRS; 41] which are standard measures and cutoffs in the bipolar literature. Participants were required to be on a stable medication regimen for at least four weeks prior to enrollment in the study and under the care of a treating physician or nurse practitioner who manages their bipolar disorder. All participants were at least 18 years old and reported English fluency, which was necessary as all aspects of the protocol were in English.

Participants also met criteria for current insomnia. Insomnia was defined as a subjective report of difficulty falling asleep (>30 minutes), difficulty maintaining sleep (wake after sleep onset >30 minutes), and/or waking up too early (early morning awakening >30 minutes), with associated daytime complaints, at least three times a week for at least one month. Further, these sleep difficulties had to occur despite adequate opportunity to sleep. These criteria reflect a combination of Research Diagnostic Criteria, [42] International Classification of Sleep Disorders [ICSD-2; 43] and DSM-IV-TR standards [15]. The Duke Structured Interview for Sleep Disorders [DSISD; 44], a valid and reliable semi-structured interview, was used to establish all insomnia diagnoses.

Exclusion criteria for the study included the following: an alcohol and/or substance abuse or dependence diagnosis within the past three months based on DSM-IV-TR criteria [15]; a current post-traumatic stress disorder diagnosis based on DSM-IV-TR criteria [15]; an active or progressive neurodegenerative disease or physical illness; evidence of sleep apnea, restless legs syndrome, or periodic limb movements during sleep; employment as an overnight shift worker in the last three months; current suicidal risk, specifically endorsements of active suicidal intent and/or a specific suicide plan; attempted suicide within the past 6 months; current homicidal risk; and pregnancy and/or breast-feeding mothers.

### Procedure

All procedures described were approved by the Committee for Protection of Human Subjects at the University of California, Berkeley and represent the pre-treatment stage of the larger NIMH-funded trial. Participants were recruited through Internet advertisements and flyers distributed to psychiatric clinics in the community. Participants were first screened over the phone to determine if they were preliminarily eligible. Once determined eligible over the phone, they were invited for a detailed in-person assessment. After written informed consent was obtained, the SCID and DSISD diagnostic interviews were administered, demographic data were recorded, and a current inter-episode state was established. The life chart was administered during the in-person assessment.

### The NIMH retrospective life-charting methodology

The National Institute of Mental Health Retrospective Life-Charting Methodology [the life chart; 45] was used to retrospectively examine the prevalence, coexistence, and persistence of sleep disturbance across a five-year period of bipolar disorder. The life chart is a validated, standard, and often utilized measure for recording retrospective features of bipolar disorder including the number, duration, and severity of mood episodes, inter-episode periods, and medication use [19,46–50] Using the life chart, we focused on the last five years because of the evidence of accurate retrospective recollection of details about life events over as much as a 5-year period [49] and the evidence that interview formats that include easy to recall anchor points are able to elicit life event information over periods ranging from 1–16 years [50].

The life chart does not include an assessment of sleep. Hence, for the present study, the life chart was carefully modified to include comorbid sleep disturbance; namely: insomnia, hypersomnia, reduced sleep need, delayed sleep phase, and irregular sleep patterns. For the purpose of the life chart, definitions of each type of sleep disturbance were based on DSM-IV-TR and ICSD-2 criteria [15,43]. Insomnia was defined as difficulty falling asleep, staying asleep, and/or waking up too early. Hypersomnia was characterized as persistent and excessive daytime sleepiness and abnormally prolonged sleep periods. Reduced sleep need was described as sleeping very little, but feeling energized despite the inadequate amount of sleep. Delayed sleep phase was defined as a circadian rhythm disorder in which the major sleep episode is two or more hours later relative to the desired bedtime, which causes difficulty waking at the desired time. Finally, irregular sleep patterns were classified as inconsistent and fragmented sleep and an erratic sleep-wake routine. In order to qualify as sleep disturbance, each type of sleep disturbance, with the exception of reduced sleep need, had to be associated with daytime complaints and/or distress.

The life chart was completed collaboratively with the participant and trained psychology doctoral students. Information about mood and sleep were collected using month-to-month units across the course of a five-year period. Previous studies have used the life chart to examine the total number of weeks ill in the past year [46]. However, since this study examined the past five years, we decided to use a less specific and easier to recall unit–months. To aid in life chart administration and participant recollection, the participant was provided with definitions and examples of mild, moderate, and severe mood episodes; definitions of the different types of sleep disturbance; and a list of life events that aided in recollection (e.g., high school graduation, loss of a job). Furthermore, participants were asked to consult diaries, medical records, doctors, friends, and family members to further ensure recollection and accuracy. Notably, a substantial proportion of participants arrived to their appointments with relevant records and associated notes.

### Analysis plan

To examine the prevalence and coexistence of sleep disturbance across bipolar disorder, the life chart data were collapsed into several variables: (a) the total number of manic, depressive, mixed and inter-episode months across the five-year period; and (b) the total number of manic, depressive, mixed and inter-episode months with comorbid insomnia, hypersomnia, reduced sleep need, delayed sleep phase and irregular sleep patterns across the same five year period. Percentages of manic, depressive, mixed, and inter-episode months characterized by each type of sleep disturbance were then calculated.

To examine the persistence of sleep disturbance across bipolar disorder, persistence was conceptualized in two ways. First, we examined the persistence of insomnia, hypersomnia, delayed sleep phase, reduced sleep need, and irregular sleep patterns across each year of the five-year period: Year 1, Year 2, Year 3, Year 4, and Year 5. Within each year, we calculated the percentage of the year that was characterized by each type of sleep disturbance. We then categorized the percentage of sleep disturbance into tertile ranges. For each year, and each type of sleep disturbance within each year, we established tertile membership. We then categorized the change in—or endurance of—sleep disturbance by examining tertile transitions from Year 1 to Year 2, Year 2 to Year 3, Year 3 to Year 4, and Year 4 to Year 5. The change in sleep disturbance was then categorized by one of the following: Developed, Persisted, Worsened, Improved, or Remitted [51]. Developed was defined as transitioning from no sleep disturbance to having sleep disturbance the following year. Persisted was defined as remaining in the same tertile categorization from one year to the next. Worsened was defined as an increase in tertile categorization from one year to the next. Improved was defined as decreasing in tertile categorization from one year to the next. Remitted was defined as transitioning from having a sleep disturbance to not having sleep disturbance the following year.

We were also interested in examining the persistence of sleep disturbance across mood episodes and inter-episode periods. To address this aim, we separately calculated the percentage of total mood months (i.e., depressed, manic, mixed) and total inter-episode months across the five year period that were characterized by each type of sleep disturbance. Paired sample t-tests were used to examine the difference between percentage of mood months and percentage of inter-episode months characterized by each type of sleep disturbance. We operationalized a non-significant t-value as indicative of considerable sleep disturbance persistence across mood episodes and inter-episode periods.

## Results

### Participant characteristics

The demographic and clinical characteristics for the 51 participants are presented in Table 1. The average age of onset for sleep disturbance was significantly earlier than the average age of onset for mood disturbance in this sample, t(50)=2.35, p=0.01.

**Figure 1:**
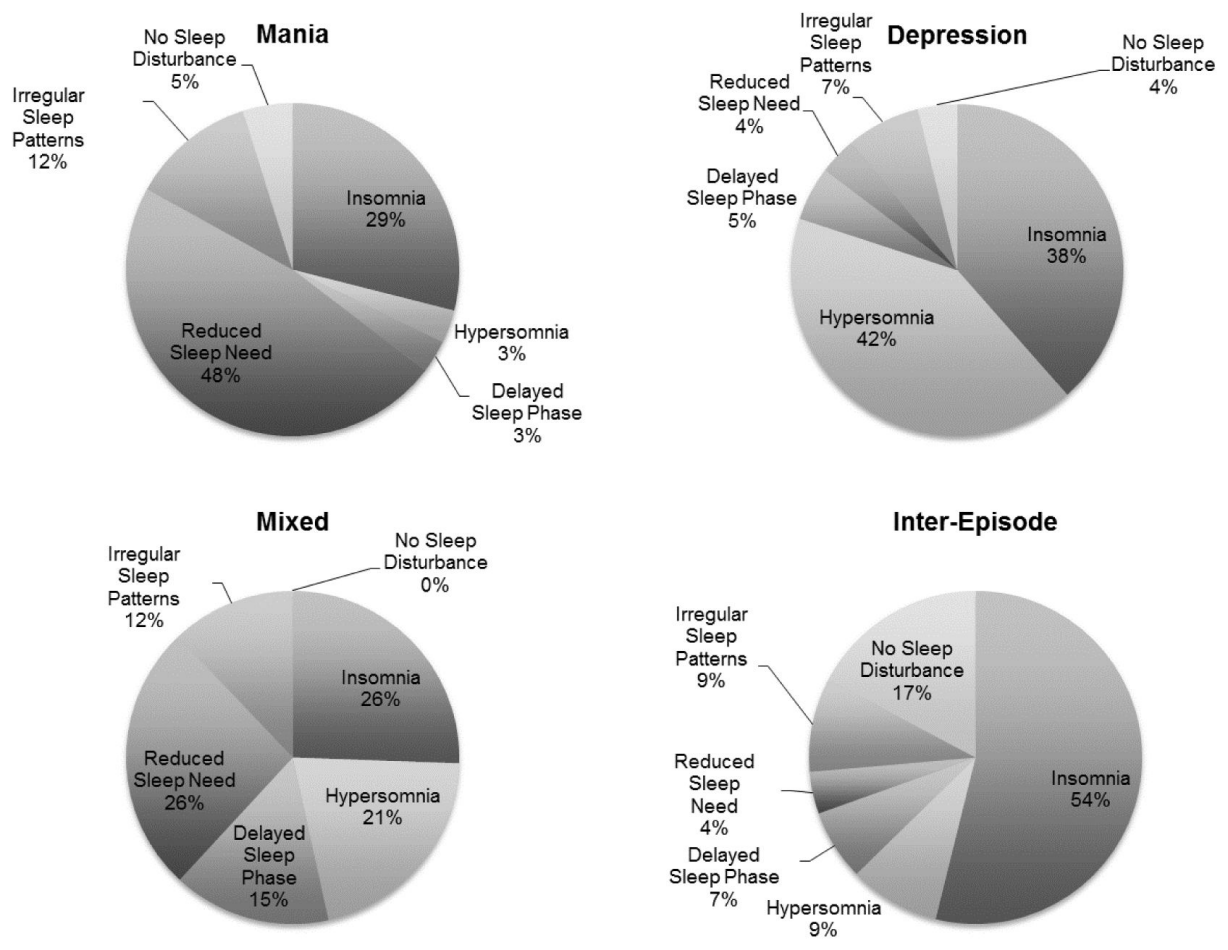
The Prevalence of Sleep Disturbance in Mood Episodes and the Inter-episode Period of Bipolar Disorder. (The percentages represent the percentage of months within each mood or inter-episode state characterized by each type of sleep disturbance).

Percentages were calculated to examine the prevalence and coexistence of insomnia, hypersomnia, delayed sleep phase, reduced sleep need, and irregular sleep patterns across mood episodes and inter-episode periods of bipolar disorder. The prevalence of each type of sleep disturbance is presented in Figure 1.

Across the course of five-years, manic months were largely characterized by reduced sleep need and insomnia. Hypersomnia and insomnia were highly prevalent during depressive months and all five types of sleep disturbance were present during mixed months. No mixed months were characterized by a lack of sleep disturbance and across manic and depressive months, having a lack of sleep disturbance was infrequent. Moving on to inter-episode months, over half of the inter-episode months were characterized by insomnia and less than a quarter was characterized by no sleep disturbance. Overall, delayed sleep phase and irregular sleep patterns were the least common features of each type of mood episode and the inter-episode period.

The coexistence of sleep disturbance is depicted in Table 2. Across mood episodes and inter-episode periods of bipolar disorder, there was coexistence in the types of sleep disturbance experienced. During manic months, the most frequently reported coexistence was for insomnia and reduced sleep need, followed by coexistence of insomnia and irregular sleep patterns. However, the percentage of months characterized by these two types of coexistence was low with percentages of 9.2% and 6.3%, respectively. Insomnia and hypersomnia coexisted in 7.9% of depressive months and 14.4% of mixed months were characterized by both hypersomnia and reduced sleep need. Lastly, inter-episode months were most prominently characterized by coexistence of insomnia and delayed sleep phase, although this percentage is small at 5.9%. The coexistence of more than two types of sleep disturbance was rare, with the exception of during mixed episode months. Mixed episode months were characterized by coexistences of: (a) insomnia, hypersomnia, and delayed sleep phase, (b) insomnia, delayed sleep phase, and reduced sleep need, and (c) insomnia, reduced sleep need, and irregular sleep patterns. The prevalence of these coexistences ranged from 6.9–8.3 %.

Persistence was operationalized in two ways. First, we were developed, persisted, worsened, improved, or remitted from year to interested in determining whether each type of sleep disturbance year. The results are presented in Table 3.

Insomnia developed in 5.8–11.8% of participants across the five years. Persistence rates for insomnia were high, ranging from 49–59%, and 3.9–13.7% of participants reported that their insomnia worsened from year to year. Finally, improvement and remission rates for insomnia were low, ranging from 3.9–11.7%. Hypersomnia developed in 5.9–19.6% of participants and persisted in 7.8–15.7% of participants. Few participants reported that their hypersomnia worsened, with percentages ranging from 2.0–5.9%. A slightly larger percentage of participants reported that their hypersomnia improved or remitted from year to year (2.0–9.8%). Delayed sleep phase was reported by a small percentage of participants. The majority of participants that reported having delayed sleep phase syndrome reported that their delayed sleep phase developed and persisted from year to year (3.9–7.8%). However, some improvement was noted as well, with 7.8% of participants reporting remission from year 2 to year 3. Reduced sleep need was primarily characterized by “developing,” “persisting,” or “remitting” from year to year. Few participants reported that their reduced sleep need worsened (3.9%) and improved (3.9%) from one year to the next. Finally, irregular sleep patterns primarily developed (1.9–13.7%) or persisted (5.9–13.7%) across the five-year period. Irregular sleep patterns also demonstrated some improvement and 5.8% of the participants reported remission from year 2 to year 3.

**Figure 2:**
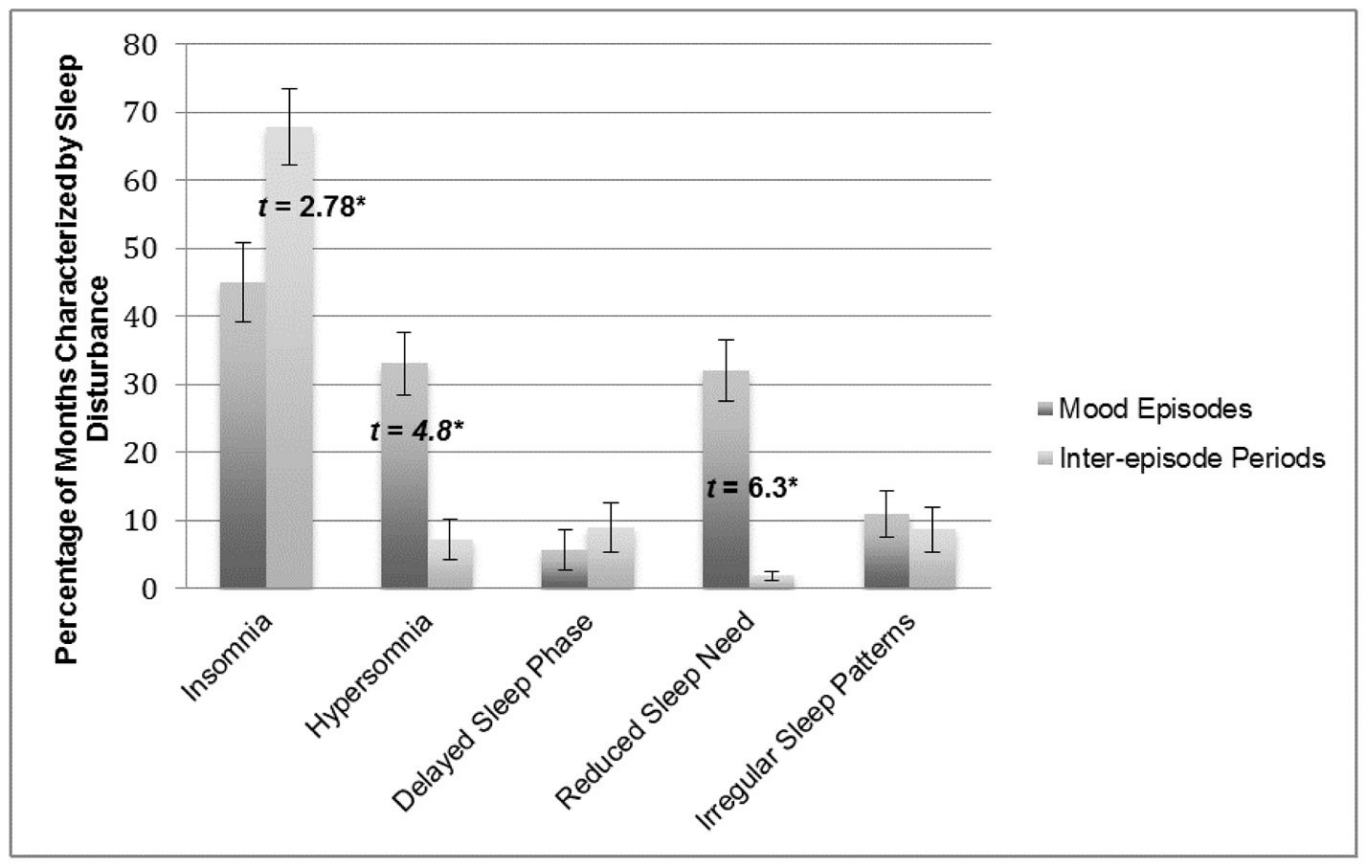
The Persistence of Sleep Disturbance: Mood Episodes vs. Inter-episode Periods. (The percentage of months within mood episodes and inter-episode periods characterized by each type of sleep disturbance are depicted in this bar graph. t-values represent the difference between mood episode and inter-episode percentage means. *p<0.01).

Second, we examined the persistence of sleep disturbance across mood episodes and inter-episode periods by conducting paired sample t-tests for each type of sleep disturbance. Non-significant t-values were conceptualized as indicative of considerable sleep disturbance persistence across mood episodes and inter-episode states. The results are presented in Figure 2.

There were significant t-values for hypersomnia and reduced sleep need, with participants reporting significantly greater hypersomnia and reduced sleep need during mood episodes compared to the inter-episode period. There was also a significant t-value for insomnia; however the percentage of insomnia during the inter-episode period was greater than the percentage of insomnia during mood episodes. T-values for both delayed sleep phase and irregular sleep patterns were not significant, pointing to the possibility that these types of sleep disturbance persist across mood episodes and inter-episode periods.

## Discussion

The present study was designed to extend knowledge about sleep and bipolar disorder by addressing the prevalence, coexistence, and persistence of sleep disturbance across a five-year period. At the outset, we wish to emphasize two important limitations. First, this study was part of a larger NIMH-funded study. Hence, all participants met diagnostic criteria for current insomnia. Given their current symptoms, we cannot rule out the possibility that participants may have exhibited biased recall of their previous sleep disturbance [e.g., 52]. Future research is needed to test whether these results are generalizable to people with bipolar disorder without current insomnia. The second limitation is that we used the NIMH Retrospective Life-Charting Methodology. Although ample recollection aids were provided, the accuracy of participant recollection cannot be verified. However, previous research demonstrates that retrospectively collecting details about life events over as much as a 5-year period is reliable [49] and that interview formats that include easy-to-recall anchor points are able to elicit life event information over periods ranging from 1–16 years [50]. Moreover, although we utilized a retrospective design, many of our results are consistent with previous cross-sectional and prospective reports that did not rely on long periods of recall [e.g., 10,16,22,53]. Nonetheless, longitudinal studies are needed. Given the limitations of this study, these results should be interpreted with caution. Nonetheless, to the best of our knowledge, this is the first study to examine the course of sleep disturbance across bipolar disorder.

Not surprisingly, results of this study demonstrated that sleep disturbance is a prevalent feature across bipolar disorder. Consistent with previous research, we found that 62.8% of manic months were characterized by reduced sleep need [16,54,55]. Interestingly, insomnia occurred in 38.1% of manic months. Whereas some studies have reported sleep continuity problems during mania, [55,56,57] to the best of our knowledge, the present study is the first to suggest that distress or impairment is associated with these sleep problems. Recall that sleep discontinuity and distress or impairment are required for a diagnosis of insomnia [15,43]. Depressive months were largely characterized by hypersomnia and insomnia, which is consistent with prior work [11,58]. Sleep during mixed episodes has been less-extensively characterized with only one study reporting that mixed episodes were associated with decreases in total sleep time [16]. Building on this study, the present results suggest that the percentage of mixed months characterized by insomnia, hypersomnia, delayed sleep phase, reduced sleep need, and irregular sleep patterns were roughly similar, with insomnia and reduced sleep need being at the upper end of the range at approximately 42%, and irregular sleep patterns being at the lower end of the range at 19.7%. Moreover, in contrast to manic, depressed, and inter-episode months, months spent in mixed states were characterized by sleep disturbance 100% of the time. Lastly, across the course of the disorder and consistent with the literature, [10,53] over two thirds of inter-episode months were characterized by insomnia.

Importantly, there was coexistence in the types of sleep disturbance experienced across manic, depressed, mixed, and inter-episode months. Approximately 10% of manic months were characterized by both reduced sleep need and insomnia. This is a novel finding. These data suggest that in addition to experiencing reduced sleep need during mania, a subset of people also experience problems initiating and/or maintaining sleep and have associated daytime complaints. Depressed months were predominantly characterized by a coexistence of insomnia and hypersomnia (7.9%), a finding that is consistent with the depression literature [22]. The most notable coexistence during mixed months was the comorbidity of hypersomnia and reduced sleep need (14.4%). A possible explanation for this finding is that perhaps within the same month, people are experiencing a decrease in the amount of sleep they are receiving (i.e., reduced sleep need) and try to compensate for this sleep loss by spending an excessive amount of time in bed [59]. Further, mixed episode months were characterized by having more than two types of sleep disturbance within a single month. These findings coupled with the fact that mixed months were characterized by sleep disturbance 100% of the time, demonstrate that sleep disturbance is a prevalent and complicated feature of mixed states. Coexistence of sleep disturbance during inter-episode months was rare. The highest coexistence was insomnia and delayed sleep phase, reported in 5.9% of inter-episode months.

There was considerable variation in the pattern of sleep disturbance across the five-year period. Notably, insomnia was the most persistent with rates ranging from 49.0–58.8%. Consistent with prior work, [26] these percentages demonstrate that insomnia is a persistent disorder with comparatively low rates of improvement and remittance (3.9–11.8%). Remarkably, even though this was an insomnia sample, a notable percentage of participants reported experiencing hypersomnia, reduced sleep need, delayed sleep phase, and irregular sleep patterns across the five-year period. The pattern of endurance and/or change varied depending on the type of sleep disturbance. However, for every type of sleep disturbance, a greater percentage of participants reported that their sleep disturbance developed or persisted compared to improved or remitted from one year to the next.

An examination of persistence across mood episodes and inter-episode periods revealed that hypersomnia and reduced sleep need are not persistent and are more commonly reported during mood episodes. This suggests that hypersomnia and reduced sleep need may be more mood state-dependent. On the other hand, there wasn’t a significant difference between delayed sleep phase and irregular sleep patterns across mood episodes and inter-episode periods, pointing to the possibility that these two types of sleep disturbance are more persistent, regardless of mood-state. Interestingly, insomnia was reported more frequently during the inter-episode period than during mood episodes. This may be a product of our sample and this finding requires replication. The assumption that non-significance is indicative of persistence requires validation.

A particularly noteworthy finding was that the average age of onset for sleep disturbance was significantly earlier than the average age of onset for mood disturbance. There is a parallel finding that is quite robust in unipolar depression. Multiple longitudinal studies have reported that sleep disturbance often precedes episodes of depression and may be a risk factor for first and recurrent episodes of depression [25, 60–62]. The current findings extend these results to bipolar disorder and suggest that sleep disturbance may be an early marker for bipolar disorder and an ideal target for early intervention. Using electroencephalogram [63,64], self-report measures, and clinical interviewing, it is possible that we can identify sleep disturbance early and intervene to improve to improve quality of life.

In sum, these findings demonstrate that sleep disturbance is a prevalent and complex feature across mood episodes and inter-episode periods of bipolar disorder. Some particularly noteworthy results include the findings that mixed episodes are characterized by sleep disturbance 100% of the time, 10% of manic months are characterized by both reduced sleep need and insomnia, and sleep disturbance tended to develop or persist over time and across mood–to–inter-episode states. These results have important implications. Due to the unequivocal relationship between sleep disturbance and mood disruption in bipolar disorder [31–37], it is possible that by further understanding the nature of sleep disturbance across the course of bipolar disorder, we can better prevent relapse into a mood episode and improve quality of life. In light of these results, it is recommended that current treatments for sleep disturbance, that encompass the complexities uncovered, should be adapted for use in bipolar disorder [58,65].

## Figures and Tables

**Table 1:** Demographic and Clinical Characteristics of People with Bipolar Disorder.

Gender (% female)	60.8% female

Employment	17.90%
Full-time (% of participants)	28.6 %
Part-time (% of participants)	53.60%
Unemployed (% of participants)	

Age (Years)	Mean ± SD 36.5 ± 11.1

Education (Years)	15.5 ± 3.7

Age at Onset Bipolar Disorder (Years)	21.2 ± 9.0

Age at Onset Sleep Disturbance (Years)	18.3 ± 9.7

Number of Manic Episodes Across the Five Year Period	1.5 ± 1.5

Duration of Manic Episodes Across the Five Year Period (Months)	2.5 ± 2.2

Number of Depressed Episodes Across the Five Year Period	1.9 ± 2.1

Duration of Depressed Episodes Across the Five Year Period (Months)	4.2 ± 3.5

Number of Mixed Episodes Across the Five Year Period	0.5 ± 1.3

Duration of Mixed Episodes Across the Five Year Period (Months)	3.0 ± 1.3

Number of Mood-Related Hospitalizations Across the Five Year Period	0.5 ± 0.8

**Table 2:** The Prevalence and Coexistence of Sleep Disturbance across Mood Episodes and Inter-episode Periods of Bipolar Disorder. (The percentages represent the percentage of months within each mood or inter-episode period characterized by each type of sleep disturbance. DELAY = Delayed Sleep Phase; REDUC = Reduced Sleep Need; IRREG = Irregular Sleep Patterns).

Sleep Disturbance	Mania	Depression	Mixed	Inter-Episode
	Mean % ± SD	Mean % ± SD	Mean % ± SD	Mean % ± SD
Insomnia Alone	16.5 ± 33.8	29.7 ± 42.0	16.7 ± 38.9	55.4 ± 43.8
Hypersomnia Alone	1.6 ± 7.2	39.2 ± 43.6	10.0 ± 28.9	6.0 ± 22.4
DELAY Alone	--	--	8.3 ± 28.9	0.5 ± 2.7
REDUC Alone	47.3 ± 44.9	0.9 ± 2.9	12.8 ± 29.5	1.0 ± 3.6
IIRREG Alone	0.8 ± 3.3	0.9 ± 3.9	12.8 ± 29.5	2.8 ± 11.9
Insomnia & Hypersomnia	--	7.9 ± 25.4	1.4 ± 4.8	0.5 ± 2.9
Insomnia & DELAY	2.8 ± 16.7	4.0 ± 14.6	--	5.9 ± 21.5
Insomnia & REDUC	9.2 ± 28.3	0.2 ± 0.9	--	0.3 ± 1.8
Insomnia & IRREG	6.3 ± 23.5	2.9 ± 16.9	--	3.2 ± 13.1
Hypersomnia & DSP	--	--	--	--
Hypersomnia & REDUC	0.4 ± 2.4	0.5 ± 2.3	14.4 ± 30.5	--
Hypersomnia & IRREG	--	1.3 ± 7.3	--	--
DELAY & REDUC	--	--	--	--
DELAY & IRREG	1.4 ± 8.3	0.1 ± 0.7	--	0.1 ± 0.9
REDUC & IRREG	3.5 ± 14.7	0.3 ± 1.7	--	--
Insomnia, Hypersomnia, & DELAY	--	2.8 ± 14.0	8.3 ± 28.9	0.1 ± 0.6
Insomnia, Hypersomnia, & REDUC	--	--	--	--
Insomnia, Hypersomnia, & IRREG	1.4 ± 6.3	1.4 ± 6.0	--	--
Insomnia, DELAY, & REDUCb	--	0.2 ± 1.4	8.3 ± 28.9	--
Insomnia, DELAY, & IRREG	--	--	--	2.1 ± 14.6
Insomnia, REDUC, and IRREG	1.7 ± 7.1	--	6.9 ± 24.1	--
Hypersomnia, DELAY, & REDUC	--	--	--	--
Hypersomnia, DELAY, & IRREG	--	--	--	--
Hypersomnia, REDUC, & IRREG	0.5 ± 3.0	--	--	0.4 ± 2.9
REDUC, DELAY, & IRREG	--	--	--	--
Insomnia, Hypersomnia, DELAY, & IRREG	--	--	--	--
Insomnia, Hypersomnia, DELAY, & REDUC	--	--	--	--
Insomnia, Hypersomnia, REDUC, & IRREG	0.3 ± 1.5	2.9 ± 16.9	--	--
Insomnia, DELAY, REDUC, & IRREG	--	--	--	--
Hypersomnia, DELAY, REDUC, & IRREG	--	--	--	--
Insomnia, Hypersomnia, DELAY, REDUC & IRREG	--	--	--	--

**Table 3:** The Persistence of Sleep Disturbance Across a Five-Year Period in Bipolar Disorder. (The percentages represent the percentage of participants within each year that experienced either a developing, persisting, worsening, improving, or remitting of each type of sleep disturbance).

Sleep Disturbance	Year 1 – Year 2	Year 2 – Year 3	Year 3 – Year 4	Year 4 – Year 5
	% of participants	% of participants	% of participants	% of participants
**Insomnia**				
Developed	9.8	5.9	9.8	11.8
Persisted	56.9	49	58.8	58.8
Worsened	5.9	3.9	9.8	13.7
Improved	3.9	11.8	3.9	3.9
Remitted	11.8	3.9	5.9	5.9
**Hypersomnia**				
Developed	5.9	11.8	11.8	19.6
Persisted	9.8	11.8	15.7	7.8
Worsened	3.9	3.9	2	5.9
Improved	2	2	3.9	9.8
Remitted	3.9	3.9	7.8	9.8
**Delayed Sleep Phase**				
Developed	5.9	3.9	5.9	3.9
Persisted	7.8	5.9	3.9	7.8
Worsened	0	0	2	2
Improved	0	0	0	0
Remitted	0	7.8	3.9	2
**Reduced Sleep Need**				
Developed	9.8	9.8	11.8	15.7
Persisted	7.8	5.9	7.8	5.9
Worsened	0	3.9	0	3.9
Improved	0	3.9	0	3.9
Remitted	15.7	3.9	15.7	5.9
**Irregular Sleep Patterns**				
Developed	7.8	2	2	13.7
Persisted	11.8	13.7	9.8	5.9
Worsened	2	0	2	3.9
Improved	0	2	2	3.9
Remitted	2	5.9	3.9	2
